# Fc gamma receptor IIIa polymorphisms in advanced colorectal cancer patients correlated with response to anti-EGFR antibodies and clinical outcome

**DOI:** 10.1186/1479-5876-10-232

**Published:** 2012-11-21

**Authors:** Rosa Calemma, Alessandro Ottaiano, Anna Maria Trotta, Guglielmo Nasti, Carmela Romano, Maria Napolitano, Domenico Galati, Pasquale Borrelli, Serena Zanotta, Antonino Cassata, Giuseppe Castello, Vincenzo Rosario Iaffaioli, Stefania Scala

**Affiliations:** 1 Oncological Immunology, National Cancer Institute “G. Pascale”, via M. Semmola, Naples 80131, Italy; 2 Abdominal Oncology, National Cancer Institute “G. Pascale”, via M. Semmola, Naples, 80131, Italy; 3 CROM - Centro Ricerche Oncologiche di Mercogliano, "Fiorentino Lo Vuolo", Via Ammiraglio Bianco, Mercogliano, (AV), Italy

**Keywords:** Fc gamma receptor, Colorectal cancer, Prognosis, Cetuximab, Panitumumab, Antibody-dependent cell-mediated cytotoxicity

## Abstract

**Background:**

Anti-EGFR monoclonal antibodies have shown efficacy in the treatment of metastatic colorectal cancer (mCRC). One of the mechanism is the antibody-dependent cell-mediated cytotoxicity (ADCC) in which Fc region of the antibody binds to the Fc gamma receptors (FcγR) expressed by immune cells. The present study investigated the association between single nucleotide polymorphisms of FcγRIIa and FcγRIIIa and clinical outcome in mCRC treated with anti-EGFR antibodies.

**Methods:**

Seventy-four consecutive patients with mCRC were analyzed. The genotypes for FcγRIIa-131 histidine (H)/arginine (R), FcγRIIIa-158 valine (V)/phenylanaline (F) polymorphisms were evaluated by directly sequencing. Multiplex allele-specific polymerase chain reaction was performed for FcγRIIIa-158 valine (V)/phenylanaline (F). Correlations between FcγR polymorphisms, baseline patient and tumor features were studied by contingency tables and the chi-square test. The Kaplan-Meier product limit method was applied to the progression-free survival (PFS) curves. Univariate analysis was performed with the log-rank test. Cox proportional-hazards regression was used to analyze the effect of multiple risk factors on PFS.

**Results:**

FcγRIIIa polymorphisms were significantly associated with response to anti-EGFR-based therapy in 49 patients with kras wt tumors (p=0.035). There was not association with response for FcγRIIa polymorphisms. Furthermore, obtained results suggested that prognosis is particularly unfavorable for patients carrying the FcγRIIIa-158F/F genotype (median PFS V/V, V/F, F/F: 18.2 vs 17.3 vs 9.4 months). No prognostic ability was identified for FcγRIIa polymorphisms.

**Conclusions:**

In mCRC patients the presence of FcγRIIIa-F can predict resistance to anti-EGFR therapy and unfavorable prognosis.

## Background

Metastatic colorectal cancer (mCRC) is the second most common cause of cancer death in the Western world accounting for 40-50% of newly diagnosed patients [1]. Despite therapeutic advances, the prognosis for patients with mCRC remains poor. However, the addition of drugs such as irinotecan and oxaliplatin to 5-fluorouracil (5-FU) has almost doubled the median survival from 12 months to 21 months [2]. Monoclonal antibodies (mAbs) binding to the vascular endothelial growth factor (VEGF) (bevacizumab) or the epidermal growth factor receptor (EGFR) (cetuximab and panitumumab) have shown efficacy in the treatment of mCRC increasing the life expectancy of patients by more than 2 years [3]. While bevacizumab is administered in combination with chemotherapy as a first-line treatment, anti-EGFR mAbs find place in later-line treatments.

Cetuximab is an IgG1a chimeric mAb while panitumumab is a fully human IgG2 mAb; they bind to EGFR and block the binding of its natural ligands, preventing ligand dependent homodimerization and activation of intracellular cascades that control cellular proliferation, adhesion, angiogenesis, and apoptosis. Anti-EGFR mAbs have proven to be effective in combination with chemotherapy or as single agents for treatment of mCRC [3]. Recent evidences showed that mCRC responds differently to EGFR-targeted agents on genetic basis that involve also the EGFR downstream effectors (i.e. kras, braf, PIK3CA and PTEN) [4]. Although largely unexplored, monoclonal antibodies also induce antibody-dependent cell-mediated cytotoxicity (ADCC) [5-8]. ADCC is induced through the interaction of the Fc region of the mAb with the Fc gamma receptor (FcγR) expressed by effector cells (i.e. natural killer-NK-lymphocytes, monocytes/macrophages). Polymorphisms have been demonstrated on genes encoding for the activating receptors FcγRIIa (CD32, mainly expressed on macrophages) and FcγRIIIa (CD16, expressed on NK cells and macrophages) [9], affecting their affinity to human IgG: a histidine (H)/arginine (R) polymorphism at position 131 for FcγRIIa and a valine (V)/phenylalanine (F) polymorphism at position 158 for FcγRIIIa. Based on the different affinities, patients harboring FcγRIIa-131H/H and FcγRIIIa-4 158V/V genotypes would be expected to mediate a more efficient ADCC antitumor response. Clinical studies utilizing rituximab in the treatment of B-cell non-Hodgkin’s lymphoma have shown that FcγRIIa-131H/H and FcγRIIIa-158V/V genotypes were associated with better clinical outcome [10,11]. Patients with 158V/V and/or 131 H/H had a significantly higher response rate than patients without either genotype (59% vs 18%). The progression-free survival (PFS) estimate of patients with 158V/V and/or 131H/H allele was also significantly longer, with median PFS of 445 and 140 days for the two groups, respectively [11]. Nevertheless it was shown that when CT is added to Rituximab the predictive value of FCGR polymorphisms was lost probably due to the high efficacy of CT [12]. In trastuzumab-treated metastatic breast cancer, ADCC analysis showed that the combination of 158 V/V and/or 131 H/H had a significantly higher trastuzumab-mediated cytotoxicity than other genotypes in addition to higher response rate and a longer PFS [13]. Contrasting results have been reported on the role of FcγR polymorphisms in mCRC [14,15]. Recently, it was described that FcγRIIa-131H/H and FcγRIIIa-158F/F polymorphisms associated with better PFS in a series of EGFR-expressing mCRC patients treated with single-agent cetuximab [14]. Conversely, Bibeau et al. demonstrated a favourable effect on PFS only for the FcγRIIIA-158V/V genotype unrelated to the kras status [15]. The goal of our study was to explore the association between FcγRIIa and FcγRIIIa polymorphisms and the outcome of mCRC patients treated with anti-EGFR-based therapies (cetuximab and panitumumab).

## Methods

### Patient management and follow-up

Seventy-four stage IV CRC patients were studied at the Division of Abdominal Medical Oncology of the National Cancer Institute (Naples, Italy) from May 2007 to May 2009. Patients were eligible after specific discussion on the study. Informed consent from each patient was sought. The protocol was conducted according to a protocol approved by the institutional review board/independent ethics committee. Patients were routinely characterized for kras mutational status [16]. All patients underwent to sequential standard treatments based on chemotherapy and/or biologic therapies (bevacizumab, cetuximab, panitumumab). First and second-line chemotherapy (CT) included the association of fluoropyrimidines (capecitabine or 5-fluoruracile) with oxaliplatin or irinotecan. The chemotherapy regimen was based on patient's performance status, extent of disease, comorbidities, previous treatments and individual preferences. Some selected patients underwent pulmonary and/or liver metastasectomies as established in a multidisciplinary team discussion. Bone metastases were treated with palliative radiotherapy. Patients features are shown in Table 1. Cetuximab or panitumumab were administered only in patients with kras wilde-type (wt) tumors. All patients underwent first-line chemotherapy, 54 patients (72.9%) received a second-line chemotherapy and 22 (29.7%) a third-line chemotherapy. Eight patients received palliative radiotherapy. Eleven patients with advanced disease underwent to palliative resection of primary colonic tumor. Metastasectomies before or after chemotherapy were performed in 26 patients. Total body computed tomography scan and CEA monitoring were done every three months. The response to therapy was evaluated by RECIST criteria. Patients with target metastatic lesions restaged at the Radiology Unit were considered for response evaluation.

**Table 1:** Detailed characteristics of patients and tumors

**Patient Initials**	**Gender**	**Age (years)**	**Mucinous component>50%**	**Primary tumor**	**Grading**	**Stage at diagnosis**	**pT**	**FcgRIIIA**	**FcgRIIA**	**PFS (months)**	**First-line CT**	**Response to first-line CT**	**Anti-EGFR therapy**	**Response to anti-EGFR therapy**
IN	Female	57	Yes	Colon	3	4	3	158V/V (G/G)	131H/H (A/A)	20,0	FU+IRI+BEV	PR	CET	PR
MM	Female	50	No	Rectum	3	4	NA	158V/F (G/T)	131H/R (A/G)	12,1	CAPE+OXA+BEV	NA	CET	SD
SU	Male	38	No	Rectum	2	3	2	158V/V (G/G)	131H/R (A/G)	17,9	FU+IRI+BEV	PR	CET	PR
RAM	Female	70	Yes	Rectum	2	1	2	158V/F (G/T)	131H/H (A/A)	10,2	FU+OXA+BEV	CR	CET	SD
AA	Female	77	No	Colon	3	4	3	158V/F (G/T)	131H/H (A/A)	6,9	FU+OXA+BEV	SD	CET	SD
MC	Male	67	No	Rectum	3	4	4	158V/V (G/G)	131H/R (A/G)	6,6	CAPE+OXA+BEV	PD	CET	SD
MU	Male	82	NA	Colon	2	3	3	158V/V (G/G)	131H/R (A/G)	NP	CAPE+OXA+BEV	SD	No	NA
AN	Female	76	No	Colon	2	4	NA	158V/V (G/G)	131H/H (A/A)	NP	CAPE+OXA+BEV	PR	No	NA
VA	Female	60	No	Colon	3	4	NA	158V/F(G/T)	131H/R (A/G)	12,1	CAPE+OXA+BEV	PR	No	NA
GA	Female	70	No	Colon	3	4	4	158V/V (G/G)	131H/R (A/G)	18,2	CAPE+OXA	PR	CET	SD
SA	Female	62	NA	Rectum	3	4	3	158V/F (G/T)	131H/R (A/G)	6,4	FU+IRI+BEV	PD	CET	PD
PG	Male	75	No	Colon	3	1	2	158F/F (T/T)	131H/R (A/G)	14,8	FU+OXA	PR	PAN	PR
LRB	Male	61	No	Colon	1	3	3	158F/F (T/T)	131H/R (A/G)	7,7	FU+IRI+BEV	SD	CET	SD
VA	Male	71	No	Colon	2	4	NA	158V/V (G/G)	131H/R (A/G)	8,8	CAPE+OXA+BEV	PD	No	NA
SG	Male	55	No	Colon	2	4	NA	158V/V (G/G)	131H/H (A/A)	19,7	FU+IRI+BEV	PD	No	NA
MR	Female	61	No	Colon	3	4	NA	158V/V (G/G)	131H/R (A/G)	10,8	CAPE+OXA+BEV	SD	No	NA
MMG	Female	43	No	Colon	2	3	4	158V/V (G/G)	131H/H (A/A)	16,1	FU+OXA+BEV	PR	PAN	PR
MM	Male	74	No	Colon	2	4	3	158V/F (G/T)	131H/H (A/A)	NP	FU+IRI+BEV	SD	No	NA
GM	Male	72	No	Rectum	3	4	NA	158F/F (T/T)	131H/R (A/G)	2,6	CAPE+BEV	SD	PAN	NA
LCR	Male	74	No	Rectum	3	3	3	158V/V (G/G)	131H/R (A/G)	23,0	CAPE	CR	CET	PR
BG	Female	47	No	Colon	3	4	NA	158F/F (G/T)	131H/R (A/G)	NP	CAPE+OXA+BEV	CR	No	NA
LG	Female	56	No	Colon	3	3	3	158V/V (G/G)	131H/R (A/G)	22,4	FU+IRI+BEV	PD	CET	SD
DMV	Male	65	No	Colon	2	3	3	158V/F (G/T)	131H/R (A/G)	22,8	CAPE+OXA+BEV	PR	CET	PR
FA	Female	66	No	Colon	2	4	4	158V/F (G/T)	131H/H (A/A)	10,0	FU+IRI+BEV	PR	CET	PR
GF	Male	56	No	Colon	3	4	NA	158V/F (G/T)	131H/H (A/A)	NP	FU+IRI+BEV	PR	No	NA
MA	Female	55	No	Rectum	2	4	NA	158V/F (G/T)	131H/H (A/A)	NP	CAPE+OXA+BEV	PR	No	NA
MMR	Female	54	No	Rectum	2	1	2	158V/F (G/T)	131H/H (A/A)	NA	FU+IRI+BEV	SD	No	NA
LS	Female	58	No	Colon	3	1	NA	158V/F (G/T)	131H/R (A/G)	15,6	FU+IRI+BEV	PR	CET	PR
CD	Male	64	No	Rectum	3	4	NA	158V/F (G/T)	131H/R (A/G)	7,0	FU+IRI+BEV	CR	CET	PR
VE	Female	56	No	Colon	2	3	3	158V/F (G/T)	131H/H (A/A)	38,0	FU+IRI+BEV	PR	CET	PR
VA	Male	81	No	Colon	2	4	NA	158V/V (G/G)	131H/R (A/G)	8,9	FU+IRI+BEV	PR	CET	SD
CM	Female	75	No	Colon	2	2	3	158V/F (G/T)	131H/H (A/A)	9,8	CAPE+OXA+BEV	SD	PAN	PR
IG	Male	73	No	Colon	3	3	NA	158V/F (G/T)	131H/H (A/A)	19,5	FU+IRI+BEV	SD	CET	CR
DGS	Male	63	No	Rectum	1	4	NA	158V/V (G/G)	131H/R (A/G)	7,7	CAPE+OXA	CR	CET	PD
RO	Female	47	No	Rectum	2	3	4	158V/F (G/T)	131R/R(G/G)	9,2	CAPE+OXA	PR	CET	SD
VA	Male	79	No	Colon	2	4	NA	158V/V (G/G)	131H/R (A/G)	13,3	FU+IRI+BEV	PR	CET	SD
DFC	Male	64	NA	Colon	2	4	3	158V/F (G/T)	131H/R (A/G)	21,3	CAPE+BEV	PR	CET	SD
EI	Female	64	No	Colon	2	4	NA	158V/F (G/T)	131H/H (A/A)	16,2	CAPE+OXA+BEV	PR	No	NA
DAR	Female	66	Yes	Colon	2	4	NA	158V/F(G/T)	131H/H (A/A)	NP	CAPE+OXA+BEV	CR	No	NA
BT	Male	56	No	Rectum	2	4	NA	158V/V (G/G)	131H/R (A/G)	NP	FU+IRI+BEV	PR	No	NA
CAG	Female	54	No	Rectum	2	3	4	158V/V (G/G)	131H/H (A/A)	11,7	CAPE+IRI+BEV	PR	CET	PR
DV	Female	78	No	Colon	3	3	3	158V/F (G/T)	131H/R (A/G)	43,6	FU+IRI+BEV	PR	CET	PR
BG	Male	65	No	Colon	3	4	NA	158V/F (G/T)	131H/H (A/A)	18,3	FU+IRI+BEV	SD	CET	PR
NG	Male	80	No	Colon	2	4	NA	158F/F (T/T)	131H/H (A/A)	5,4	FU	PD	No	NA
NR	Female	42	No	Colon	3	4	NA	158V/F(G/T)	131H/R (A/G)	6,7	CAPE+OXA+BEV	PD	No	NA
MR	Male	60	No	Colon	3	4	3	158V/F (G/T)	131H/R (A/G)	8,7	FU+IRI+BEV	PR	CET	SD
SF	Female	62	No	Rectum	2	4	NA	158V/F (G/T)	131R/R(G/G)	14,2	CAPE	SD	CET	SD
DCM	Male	60	No	Colon	2	4	NA	158V/F (G/T)	131H/R (A/G)	37,5	FU+IRI+BEV	PR	CET	PR
CML	Female	44	No	Colon	3	3	3	158V/F (G/T)	131H/R (A/G)	29,3	CAPE+OXA+BEV	CR	CET	PR
BM	Male	66	No	Rectum	3	4	NA	158V/V (G/G)	131H/R (A/G)	8,1	CAPE+BEV	SD	CET	SD
MI	Female	40	NA	Colon	2	4	NA	158V/V (G/G)	131H/R (A/G)	NP	FU+IRI+BEV	PR	No	NA
GC	Female	57	No	Rectum	2	4	NA	158V/V (G/G)	131H/H (A/A)	NP	CAPE+OXA+BEV	PR	No	NA
SC	Male	53	No	Rectum	3	4	NA	158V/V (G/G)	131H/R (A/G)	NP	CAPE+OXA+BEV	SD	No	NA
RR	Male	77	No	Colon	2	2	1	158V/F (G/T)	131H/R (A/G)	9,4	FU+OXA+BEV	PR	PAN	SD
GA	Female	70	No	Colon	3	4	4	158V/V (G/G)	131H/R (A/G)	14,6	CAPE+OXA	PR	No	SD
MP	Male	78	No	Colon	3	4	3	158V/F (G/T)	131H/R (A/G)	9,6	CAPE+OXA+CET	SD	CET	SD
CM	Male	67	No	Rectum	3	3	3	158V/V (G/G)	131H/H (A/A)	43,6	IRI+CET	CR	CET	SD
FR	Female	55	Yes	Rectum	3	4	NA	158V/V (G/G)	131R/R(G/G)	12,4	CAPE+OXA	SD	CET	SD
GL	Male	74	Yes	Colon	2	4	NA	158V/V (G/G)	131H/H (A/A)	NP	FU+OXA+BEV	PR	No	NA
TMA	Female	66	No	Colon	1	4	NA	158V/V (G/G)	131H/R (A/G)	NP	FU+IRI+BEV	PR	No	NA
CF	Female	71	NA	Rectum	2	4	NA	158V/V (G/G)	131H/R (A/G)	NP	CAPE+IRI+BEV	PR	No	NA
CE	Female	55	No	Colon	1	1	4	158V/V (G/G)	131R/R(G/G)	6,1	CAPE+IRI+CET	SD	CET	SD
SME	Male	63	No	Colon	3	4	4	158V/V (G/G)	131H/R (A/G)	NP	CAPE+IRI+CET	PR	CET	PR
GM	Male	56	No	Rectum	3	4	4	158F/F (T/T)	131H/H (A/A)	7,2	CAPE+OXA+CET	PR	CET	PD
IP	Male	69	No	Rectum	3	4	NA	158V/V (G/G)	131H/H (A/A)	7,8	CAPE+OXA	PR	CET	PR
TMR	Female	68	Yes	Colon	3	4	NA	158V/V (G/G)	131H/R (A/G)	8,8	CAPE+OXA+CET	PD	CET	SD
PM	Female	77	No	Colon	1	3	3	158F/F (T/T)	131H/H (A/A)	9,4	CAPE+OXA	CR	CET	PD
MG	Male	64	No	Rectum	3	4	NA	158V/F (G/T)	131H/H (A/A)	16,5	IRI+CET	PD	CET	SD
MG	Male	68	No	Colon	3	4	NA	158V/F (G/T)	131H/H (A/A)	2,8	CAPE+OXA	PD	CET	PD
PG	Male	64	No	Rectum	3	2	3	158V/F (G/T)	131H/H (A/A)	37	FU	PR	CET	SD
SS	Male	82	No	Rectum	3	4	NA	158V/F (G/T)	131H/R (A/G)	19,4	CAPE	PR	CET	PD
FP	Male	69	Yes	Colon	3	4	NA	158V/F (G/T)	131H/R (A/G)	20,8	FU+IRI	SD	CET	PR
PA	Female	70	NA	Colon	2	4	3	158V/F (G/T)	131H/H (A/A)	23,3	CAPE+OXA	CR	No	NA
DLA	Male	82	No	Colon	3	3	2	158F/F (T/T)	131H/R (A/G)	28,0	CAPE+OXA	SD	CET	PD

*CT*: Chemotherapy: *NA*: Not Assessable; *NP*: Not Progressed; *CR*: Complete Response; *PR*: Partial Response; *SD*: Stable Disease; *PD*: Progressive Disease; *PFS*: Progression Free Survival; *FU*: Fluorouracile; IRI: Irinotecan; *CAPE*: Capecitabine; *BEV*: Bevacizumab; PAN: Panitumumab; *CET*: Cetuximab.

Complete response (CR) was defined as complete disappearance of all detectable evidence of disease on total body computed tomography. Partial response (PR) was defined as at least a 30% decrease in the sum of diameters of target lesions. Stable disease (SD) was defined as everything between 30% decrease and 20% growth of tumor size. Progressive disease (PD) was defined as at least a 20% increase in the sum of diameters of target lesions. Two patients were lost at follow-up.

### Analysis of FcγRIIa-H131R, FcγRIIIa-V158F polymorphisms

**Figure 1:**
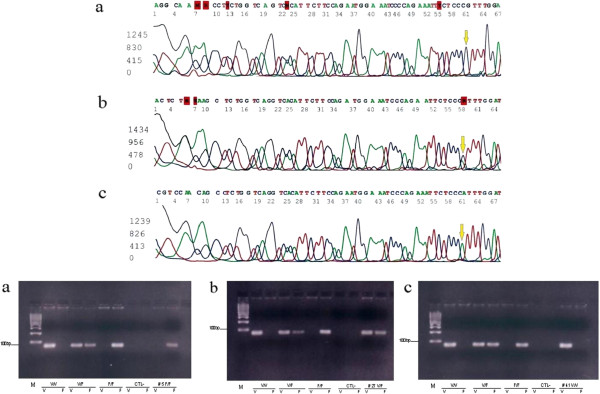
**FcγRIIa determined by direct sequencing and FcγRIIIa allotyping by allele**-**specific PCR. v Upper Panel.** FcγRIIa determined by direct sequencing: (**a**) Sequencing electropherogram obtained from a sample homozygous for allele FcγRIIa 131H/H; (**b**) Sequencing electropherogram obtained from a sample heterozygous for allele FcγRIIa 131H/R; (**c**) Sequencing electropherogram obtained from a sample homozygous for allele FcγRIIa 131R/R. **Lower Panel.** FcγRIIIa allotyping by allele-specific PCR. 100bp ladder marker, FcγRIIIa genotypes direct sequenced F/F, V/F and V/V control and CTL- negative control were represented. Examples represented respectively F/F (**a**), V/F (**b**) and V/V (**c**) patients.

Genomic DNA was extracted from white blood cells (WBCs) using a DNA extraction kit.(Qiagen,Valencia, CA) and stored at −20°. FcγRIIa genotyping was performed on genomic DNA by polymerase chain reaction (PCR) method adapted from a previously established protocol [17]. Briefly, PCR amplification was performed in 50 μl reaction mixture containing 100 ng genomic DNA, 0.4 mM of each primer, 0.4 mM dNTPs, 20 mM Tris–HCl, pH 9.0, 100 mM KCl, 0.1 mM EDTA, 1.0 mM DTT, 0.5% tween 20, and 1 U Taq DNA polymerase. The program was performed in the thermal Cycler 2770 by Applied Biosystems and consisted of an initial denaturation step at 95°C for 5′, followed by 36 cycles of 95°C for 30 seconds, 56°C for 40 seconds, 72°C for 40 seconds and a final elongation step at 72°C for 10 minutes. The primers used for PCR amplification were forward primer 5′-GGAGAAACCATCATGCTGAG-3′ and reverse primer 5’-CAATTTTGCTGCTATGGGC-3′. The resulting PCR product (289bp) was purified with the Montage SEQ_96_ Sequencing Reaction Cleanup Kits (Millipore) and prepared to sequence through a second PCR reaction using Big Dye Terminator v3.1 Cycle Sequencing Kit by Applied Biosystems in forward and reverse direction of the region of interest (96°C for 1 minutes, 25 cycle of 96°C for10 seconds, 56°C for 5 seconds and 60°C for 2 minutes). PCR product was purified with Montage SEQ_96_ Sequencing Reaction Cleanup Kits (Millipore) and direct sequencing was run with Applied Biosystems3130 Genetic Analyzers (Figure 1).

For FcγRIIIa-V158F polymorphism, allele-specific PCR method was followed. Briefly, 100 ng of genomic DNA was amplified using allele-specific common forward primer 5′-TCCAAAAGCCACACTCAAAGAC-3′ and reverse primer 5′-CTGAAGACACATTTTTACTCCCAAAC-3′. PCR amplification was performed in 25 μl reaction mixture containing 100 ng genomic DNA, 0.3 mM of each primer, 0.2 mM dNTPs, 20 mM Tris–HCl, pH 9.0, 100 mM KCl, 0.1 mM EDTA, 1.0 mM DTT, 0.5% tween 20, and 1 U Taq DNA polymerase. The program was performed in the thermal Cycler 2770 by Applied Biosystems and consisted of an initial denaturation step at 95°C for 5′, followed by 35 cycles of 94°C for 30 seconds, 64°C for 30 seconds, 72°C for 30 seconds and a final elongation step at 72°C for 10 minutes. Three DNA samples previously sequenced FcgRIIIa-V/V158, FcgRIIIa-V/F158, FcgRIIIa-F/F158 were run in all reactions (Figure 1). The reaction products were run on 3% ethidium bromide-stained agarose gel. Seventy-three base pair PCR fragment either positive for valine (V) or F allele was visualized under UV light as reported previously [18]. To confirm FcγRIIIa genotype automatic sequencing was performed using forward primer 5′- TGT AAA ACG ACG GCC AGT TCA TCA TAA TTC TGT CTT CT-3′; reverse primer 5′–CAG GAA ACA GCT ATG ACC CTT GAG TGA TGG TGA TGT TCA-3′. The part of exon 4 which contains the polymorphic site was amplified by PCR using 100 ng genomic DNA, 0.4 mM of each primer, 0.4 mM dNTPs, 20 mM Tris–HCl, pH 9.0, 100 mM KCl , 0.1 mM EDTA, 1.0 mM DTT, 0.5% tween 20, and 1 U Taq DNA polymerase. The program was performed in the thermal Cycler 2770 by Applied Biosystems and consisted of an initial denaturation step at 95°C for 5′, followed by 36 cycles of 95°C for 30 seconds, 57°C for 30 seconds, 72°C for 30 seconds and a final elongation step at 72°C for 10 minutes. The PCR product was sequenced using the Big Dye Terminator v3.1 Cycle Sequencing Kit by Applied Biosystems.

### Statistical analyses and data presentation

Associations between FcγR polymorphisms and clinical pathologic variables were evaluated by χ^2^ test. p < 0.05 was considered statistically significant. Genotype data for FcγR polymorphisms and clinic-pathological variables were retrospectively collected and associated with response to anti-EGFR-based therapy by χ^2^ test with level of significance set at p < 0.05. Progression-free survival (PFS) was defined as the time elapsed from the treatment initiation and tumor progression or death from any cause. The Kaplan-Meier product limit method was applied to graph PFS. Univariate analysis was done with the log-rank test. Cox proportional hazards regression was used to analyze the effect of several risk factors on PFS. Risk factors (covariates) were: age, sex, grading, response to I° line chemotherapy, FcγR polymorphisms. Ninety-five percent confidence intervals of hazard ratios were also reported. No attempts were done to analyze overall survival because of low events. Seventy-two patients were analyzed since two were lost at follow-up. Statistical analysis was performed using the MedCalc® 9.3.7.0 and Excel software.

## Results

### Characteristics of patients and tumors

Seventy-four patients seen from May 2007 to May 2009 were studied for the FcγRIIa and FcγRIIIa polymorphisms through direct sequencing and allele specific PCR as reported in Figure 1. Patients features are detailed in Table 1. Median age was 65 years. Genders were equally represented. Twenty-six tumors originated in the rectum 51.4% of patients had high-grade (G3) disease. The majority of lesions presented with a pT3 extent of invasion at diagnosis and 22 presented with pN+ disease. Fifty patients presented with stage IV disease, 16 with stage III and 8 with stage I/II. The majority of tumors (90.5%) did not have a mucinous component; the most represented histology was pure colonic adenocarcinoma (Table 1).

### FcγRIIIa but not FcγRIIa polymorphisms were significantly associated with response to anti-EGFR-based therapy in kras wt tumors

Fifty patients were treated with anti-EGFR-based therapy and forty nine were evaluable for clinical response (according to RECIST criteria) and PFS. Forty-five patients were treated with cetuximab, five with panitumumab. The genotypic frequencies of FcgRIIIA and FcgRIIa detected within the analyzed population were 36% VV, 54% VF ,10% FF and 36% HH, 56% HR, 8% RR, respectively. The χ^2^ test showed that there were no significant differences in the genotype frequencies (p=0.109 for V158F; p=0.183 for FcgRIIa) between patients and healthy controls. The genotypic distributions were in Hardy-Weinberg Equilibrium.

Objective responses according to FcγR polymorphisms were shown in Table 2. FcγRIIIa but not FcγRIIa polymorphisms were significantly associated with response to anti-EGFR-based therapy in kras wt tumors (p=0.035). The mean number of anti-EGFR therapy cycles were 15 (range: 5–27) considering panitumumab as single administration every two weeks (one administration=1 cycle) and cetuximab weekly (two administrations=1 cycle). To evaluate skin toxicity and its predictive role and correlation with FcgR polymorphisms (21), the skin related toxicity was evaluated versus the clinical response. A significant correlation was identified ( p= 0.005) between skin toxicity and clinical response (Table 3) while no significant correlation was identified between skin toxicity and the genotype distribution (Table 4).

**Table 2:** Response to anti-EGFR therapy according to FcγR polymorphisms

		**FcγRIIIa**			**FcγRIIa**		
	**Total no.(%)**	**V/V**	**V/F**	**F/F**	**H/H**	**H/R**	**R/R**
**Response to anti-EGFR based chemotherapy (49 KRAS-wt evaluable pts)**							
CR+PR	20 (40.8)	7	12	1	9	11	0
SD	22 (44.9)	10	11	1	5	13	4
PD	7 (14.3)	1	3	3	3	4	0
*P*		*0.035*			*0.344*		

**Table 3:** Correlation between skin toxicity and response to anti-EGFR therapy (A), and polymorphisms

	**Response to anti-EGFR therapy**				
	**CR**	**PR**	**SD**	**PD**	P**
Skin toxicity grade*					
Grade 1	0	3	7	5	
Grade 2	0	4	11	2	
Grade 3	1	12	4	0	*0.005*

**Table 4:** polymorphisms

	**V/V**	**V/F**	**F/F**	P**	**H/H**	**H/R**	**R/R**	P**
Skin toxicity grade*								
Grade 1	3	9	3		5	10	0	
Grade 2	7	8	2		5	9	3	
Grade 3	8	9	0	*0.2707*	7	9	1	*0.4198*

*According to Common Toxicity Criteria for Adverse Event v3.0 and defined as any grade of rash/acne/dermatitis.

P** at Chi-Square test.

**Table 5:** Uni- and multivariate analyses for progression-free survival (PFS)

	**Events/Patients**	**Median PFS(months)**	***P***^***1***^	**HR**^**2**^	**95% CI**^***3***^	***P***^***4***^
**Covariate**						
Age (≤70 vs >70 years)	32/36 vs 11/13	17.0 vs 18.0	0.50	0.61	0.30-1.20	0.15
Sex (male vs female)	24/28 vs 19/21	18.3 vs 15.6	0.73	1.28	0.69-2.35	0.42
Grading (G1/G2 vs G3)	15/20 vs 28/29	17.3 vs 13.3	0.007	1.83	1.01-3.31	0.04
Response to 1^st^-line CT0 (CR vs PR vs SD vs PD)	7/8 vs 18/23 vs 12/12 vs 6/6	20.1 vs 20.0 vs 9.8 vs 7.6	0.0026	1.86	1.32-2.62	0.0004
FcγRIIIa (VV vs VF vs FF)	13/18 vs 25/26 vs 5/5	18.2 vs 17.3 vs 9.4	0.04	2.35	1.37-4.01	0.001
FcγRIIa (HH vs HR vs RR)	17/18 vs 23/27vs 3/4	16.1 vs 18.2 vs 13.3	0.61	1.19	0.72-1.96	0.49

*P*^1^ = Log Rank *P*.

HR^2^ = Cox regression HR.

CI^3^ = Confidence Intervals.

*P*^4^ = Cox’s Proportional Hazards Regression *P*.

### FcγR polymorphisms predict PFS in mCRC patients treated with anti-EGFR mAbs

**Figure 2:**
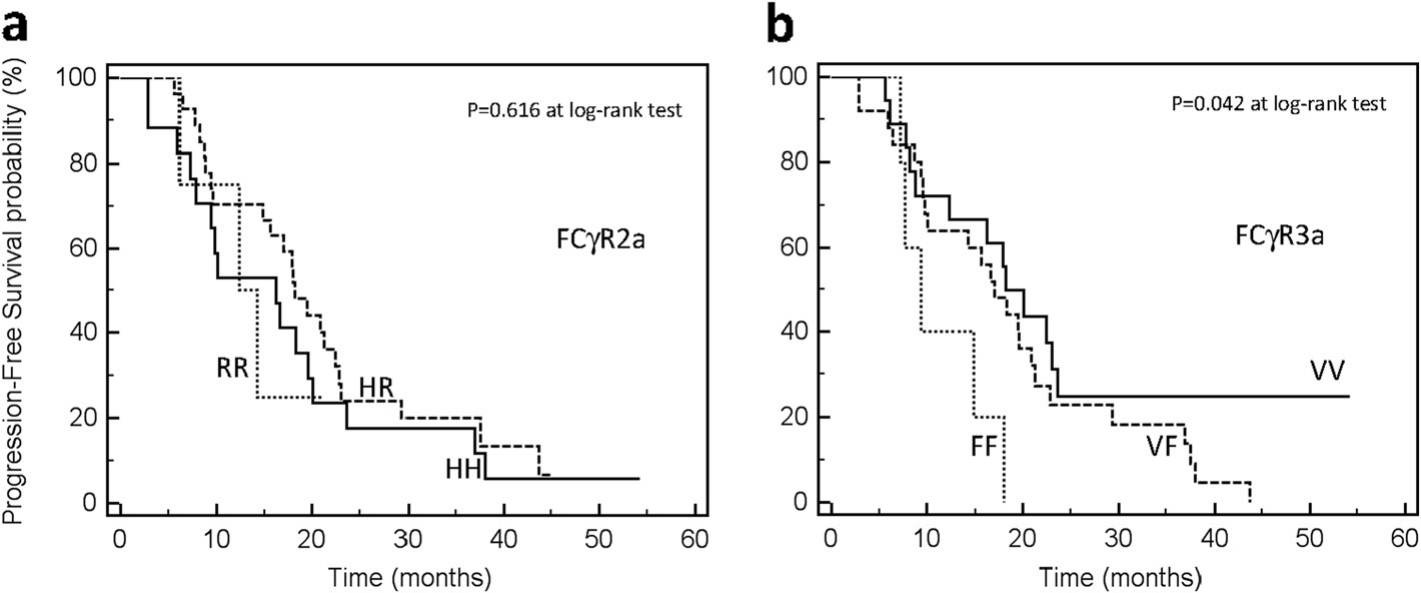
**Progression-free survival curves according to FcγR polymorphisms on 49 mCR Cpatients.** Progression-free survival was defined as time elapsed between treatment initiation and tumor progression (**a**) FcγRIIa: median PFS was 16.1 months in H/H patients (18 patients, 13 events) vs 18.2 months in H/R patients (27 patients, 23 events) vs 13.3 months in R/R patients (4 patients, 3 events); Log Rank test for three curves: p = 0.61. (**b**) FcγRIIIa: median PFS was 18.2 months in V/V patients (18 patients, 13 events) vs 17.3 months in V/F patients (26 patients, 25 events) vs 9.4 months in F/F patients (5 patients, 5 events); Log Rank test for three curves: p = 0.04.

The anti-EGFR treated patients were analyzed for PFS. As of June 2011, after a median follow-up for alive patients of 22.4 months, 43 patients (87.7%) had suffered tumor progression and 19 (44.2%) had died. Median PFS was 17.0 months. Analysis of prognostic factors for PFS is summarized in Table 5. Grading, response to 1st-line chemotherapy and FcγRIIIa polymorphisms had a significant prognostic value with univariate analysis. No prognostic ability was identified for FcγRIIa polymorphisms. The prognostic value of the grading (p=0.04, HR: 1.83, CI: 1.01-3.31), response to I°-line chemotherapy (p=0.0004,HR:1.86,CI:1.32-2.62) and FcγRIIIa (p=0.001, HR:2.35; CI:1.37-4.01) was confirmed with multivariate analysis (Table 5). Hazard ratios of relapse and pattern of Kaplan-Meier estimated curves suggest that prognosis is particularly unfavorable for patients expressing the FcγRIIIa-158F/F genotype (median PFS V/V, V/F, F/F: 18.2 vs 17.3 vs 9.4 months) (Figure 2).

## Discussion

In this manuscript the value of the FcγRIIa-FcγRIIIa polymorphisms was retrospectively correlated to the efficacy of anti-EGFR therapy in mCRC. FcγRIIIa polymorphisms were significantly associated with response to anti-EGFR-based therapy in 49 valuable patients with kras wt tumors. The results suggested that prognosis is particularly unfavorable for patients expressing the FcγRIIIa-158F/F genotype versus patients carrying a V allele (the FcγRIIIa-158F/V or the FcγRIIIa-158V/V genotypes). On this issue, conflicting results were previously described: Bibeau et al. showed a statistically significant difference in PFS in 69 mCRC patients treated with cetuximab plus irinotecan carrying the the FcγRIIIa-158V/V genotype compared to other combinations expressing an F allele while FcγRIIa polymorphisms did not affect prognosis [15]. Conversely, in a series of 39 EGFR-expressing mCRC patients treated with single-agent cetuximab, Zhang et al. [14] found that FcγRIIa-H131R and FcγRIIIa-V158F polymorphisms were independently associated with better PFS. However, against their hypothesis, FcγRIIIa-158V/V genotype was associated with more unfavorable clinical outcome. The authors suggest that variants of human IgG1–binding sites can influence the ADCC by modulating complex interactions with activating (FcγRIIIa) or inhibitory (FcγRIIb) receptors on effector cells.

In the present study a significant association between FcγRIIIa-158V/V genotype and response to anti-EGFR-based chemotherapy was demonstrated in 49 kras wt patients confirming that the expression of the allele F predicts a worse response and a shorter PFS. In the evaluated population, 49 patients, 5 (10.2%) carried the FF genotype for FcγRIII and 4 of 49 (8.2%) the RR genotype for FcγRII. These frequencies differ from those previously reported. In particular, Carlotti et al. investigated on 94 Italian patients affected by follicular lymphoma and thus treated with Rituximab; they reported 30 FF (32%) and 18 RR (19%) distribution [12]. Bibeau et al. analyzed a cohort of European subjects affected by mCRC-cetuximab treated, reporting 15 FF (22%) and 17 (28%) RR patients. Nevertheless, comparing the allelic frequencies in a population of 168 healthy donors there were no significant differences in the genotype frequencies (χ^2^ test p=0.109 for V158F; p=0.183 for FcgRIIa). Moreover we can also speculate that, although the studied population represent 74 consecutive mCRC patients coming to our observation, they all showed a good Performance Status (0–1) further validated by the neoadjuvant treatment for 23 patients (Fluopirimidine/ Irinotecan/ Bevacizumab) before hepatic metastasectomy.

The role of ADCC induced by EGFR-specific mAbs may prevent tumor outgrowth or metastasis in vivo, even in cancers insensitive to EGFR signaling inhibition [19]. In fact, evidences accumulated on a complex patients evaluation including the kras status but also EGF/EGFR polymorphisms and downstream pathway mutations. To date, unless a large number of patients have been treated with mAbs there are still two crucial issues: i) a small percent of kras mutated patients respond to cetuximab therapy [3,4] and, ii) although kras wt tumors are potentially sensitive to EGFR-targeted mAbs, not all respond to anti-EGFR therapy for multiple target expression, amplification or mutations downstream [4]. Blockade of signal transduction may not be the only mechanism of action mediating clinical benefit of mAb-treated patients with colorectal cancer [20]. Cell-dependent lysis of target cells mediated by mAbs in vitro and in animal models is a crucial mechanism of action regulated by multiple factors (i.e. FcγR on Dendritic Cells, T-helper system, Tregs, B-cells, NK-cells inhibitory proteins, cytokines, etc.). Currently, we are studying the correlation between specific FCγR polymorphisms to in vitro ADCC efficacy (Trotta et al., manuscript in preparation).

## Conclusions

Although the exact role of FcγRIIIa-V158F polymorphism and anti-EGFR therapy require more basic studies, the presence of one allele F of the FcγRIIIa in mCRC patients predicted poor response to anti-EGFR-based therapy and worsen the prognosis.

## Competing interests

The authors declare that they have no competing interests.

## Authors’ contributions

RC and AO treated the patients and carried out the molecular studies. AMT carried out the molecular studies and helped to draft the study. GN participated in its design and coordination and management of patients. CR participated in management of patients. MN carried out the molecular studies and helped to draft the study. DG carried out the molecular studies and helped to draft the study. PB carried out the molecular studies and helped to draft the study. SZ carried out the molecular studies and helped to draft the study. AC participated in its design and coordination and management of patients. GC conceived of the study. VRI conceived of the study, and participated in its design. SS conceived of the study, and participated in its design, statistical analysis and coordination and helped to draft the manuscript. All authors read and approved the final manuscript.

## References

[B1] Siegel R Ward E Brawley O Jemal A Cancer statistics, 2011: the impact of eliminating socioeconomic and racial disparities on premature cancer deaths CA Cancer J Clin 2011 61 212 236 10.3322/caac.20121 21685461

[B2] Davies JM Goldberg RM Treatment of metastatic colorectal cancer Semin Oncol 2011 38 552 5560 10.1053/j.seminoncol.2011.05.009 21810514

[B3] Tol J Punt CJ Monoclonal antibodies in the treatment of metastatic colorectal cancer: a review Clin Ther 2010 32 437 453 10.1016/j.clinthera.2010.03.012 20399983

[B4] Bardelli A Siena S Molecular mechanisms of resistance to cetuximab and panitumumab in colorectal cancer J Clin Oncol 2010 28 1254 1261 10.1200/JCO.2009.24.6116 20100961

[B5] Ottaiano A Scala S Iaffaioli RV Cetuximab-dependent ADCC in cancer: dream or reality? Cancer Immunol Immunother 2010 59 1607 1608 10.1007/s00262-010-0884-3 20577878 PMC11030127

[B6] Correale P Marra M Remondo C Cytotoxic drugs up-regulate epidermal growth factor receptor (EGFR) expression in colon cancer cells and enhance their susceptibility to EGFR-targeted antibody-dependent cell-mediated-cytotoxicity (ADCC) Eur J Cancer 2010 46 1703 1711 10.1016/j.ejca.2010.03.005 20399639

[B7] Bier H Hoffmann T Haas I van Lierop A Anti-(epidermal growth factor) receptor monoclonal antibodies for the induction of antibody-dependent cell-mediated cytotoxicity 19 against squamous cell carcinoma lines of the head and neck Cancer Immunol Immunother 1998 46 167 173 10.1007/s002620050475 9625540 PMC11037359

[B8] García-Foncillas J Díaz-Rubio E Progress in metastatic colorectal cancer: growing role of cetuximab to optimize clinical outcome Clin Transl Oncol 2010 12 533 542 10.1007/s12094-010-0551-3 20709651

[B9] McKenzie SE Schreiber AD Biological advances and clinical applications of Fc receptors for IgG Curr Opin Hematol 1994 1 45 52 9371259

[B10] Cartron G Dacheux L Salles G Therapeutic activity of humanized anti-CD2 monoclonal antibody and polymorphism in IgG Fc receptor Fc RIIIa gene Blood 2002 99 754 758 10.1182/blood.V99.3.754 11806974

[B11] Weng WK Levy R Two immunoglobulin G fragment C receptor polymorphisms independently predict response to rituximab in patients with follicular lymphoma J Clin Oncol 2003 21 3940 3947 10.1200/JCO.2003.05.013 12975461

[B12] Carlotti E Palumbo GA Oldani E FcgammaRIIIA and FcgammaRIIA polymorphisms do not predict clinical outcome of follicular non-Hodgkin's lymphoma patients treated with sequential CHOP and rituximab Haematologica 2007 92 1127 1130 10.3324/haematol.11288 17650444

[B13] Musolino A Naldi N Bortesi B Immunoglobulin G fragment C receptor polymorphisms and clinical efficacy of trastuzumab-based therapy in patients with HER-2/neu-positive metastatic breast cancer J Clin Oncol 2008 26 1789 1796 10.1200/JCO.2007.14.8957 18347005

[B14] Zhang W Gordon M Schultheis AM Fc R2A and Fc R3A Polymorphisms associated with clinical outcome of epidermal growth factor receptor–expressing metastatic colorectal cancer patients treated with single-agent cetuximab J Clin Oncol 2007 25 3712 3718 10.1200/JCO.2006.08.8021 17704420

[B15] Bibeau F Lopez-Crapez E Di Fiore F Impact of Fc RIIa-Fc RIIIa polymorphisms and KRAS mutations on the clinical outcome of patients with metastatic colorectal cancer treated with cetuximab plus irinotecan J Clin Oncol 2009 27 1122 1129 10.1200/JCO.2008.18.0463 19164213

[B16] Carotenuto P Roma C Cozzolino S Detection of KRAS mutations in colorectal cancer with Fast COLD-PCR Int J Oncol 2012 40 378 384 21971641 10.3892/ijo.2011.1221

[B17] Jiang XM Arepally G Poncz M McKenzie S Rapid detection of the FcyRIIA-H/R131 ligand-binding polymorphism using an allele-specific restriction enzyme digestion (ASRED) J Immunol Methods 1996 199 55 59 10.1016/S0022-1759(96)00164-0 8960098

[B18] de Straat FG L-v van der Pol WL Jansen MD A novel PCR-based method for direct Fc gamma receptor IIIa (CD16) allotyping J Immunol Methods 2000 242 127 132 10.1016/S0022-1759(00)00240-4 10986395

[B19] Overdijk MB Verploegen S van den Brakel JH Epidermal growth factor receptor (EGFR) antibody-induced antibody-dependent cellular cytotoxicity plays a prominent role in inhibiting tumorigenesis, even of tumor cells insensitive to EGFR signaling inhibition. J Immunol 2011 187 3383 3390 10.4049/jimmunol.1003926 21832160

[B20] Van Cutsem E Tejpar S Vanbeckevoort D Intrapatient cetuximab dose escalation in metastatic colorectal cancer according to the grade of early skin reactions: the randomized EVEREST study J Clin Oncol 2012 30 2861 2868 10.1200/JCO.2011.40.9243 22753904

